# The Seventh Aniversary of the American Medical Association

**Published:** 1854-06

**Authors:** 


*The Seventh Anniversary of the American Medical Association, held at St. Louis, Mo., May 2nd—5th*, 1854.

In the May Number of the Journal, we gave a condensed account of the business proceedings of the American Medical Association, at its recent meeting, but omitted all reference to the collateral doings and entertainments. In justice to the Profession of St. Louis, as well as to our own feelings, we recur to the subject again. To say that the Committee of Arrangements, the Profession of St Louis, and some of the most distinguished Citizens, spared no effort or expense to entertain the members of the Association, in a style betokening the most liberal hospitality, would be to speak entirely within the limits of truth. On the first evening of the Session, invitations were given to visit the residences of Ex-Mayor Kennet, and Drs. Moore, McPheeters, and Reyburn. At each of these places, almost every luxury was provided that could gratify the palate, or feast the senses, in addition to the highest degree of social enjoyment.

On the evening of the second day, the Association accepted the invitation to visit the princely residence of Col. O'Fallen, located about four miles from the central part of the city. A sufficient number of Omnibuses were provided by the Col. to take all his guests from the place of meeting to his residence. The ride was delightful, except a superabundance of dust, and the reception most cordial and pleasant. The residence occupies the top of a hill or bluff, and from the front overlooks the Mississippi, with a wide and beautiful landscape, while in the rear, is one of the most extensive lawns, decorated with flowers, evergreen shrubs, fruit trees, walks, &c., &c., which can be found in America. In the interior of the mansion, was everything that wealth and cultivated taste could procure.

Between 9 and 11 o'clock, P.M., the large company returned to the city, all apparently highly pleased with their ride, their reception, the entertainment, and above all with the generous host and patron of the profession, Col O'Fallen, himself.

On the the third and last evening, came the grand public entertainment provided by the Profession of St. Louis, in the spacious Hall of the Mercantile Library building.

Here many hundred dollars had been spent in providing one of the most splendid entertainments ever given in America, and only equalled by the one given to the Association, in New York, the year previous. The Hall was splendidly decorated, with flowers, evergreens, and mottoes, and provided with an excellent band of music. The tables, in extent sufficient to seat between 500 and 600, were literally loaded with everything that man could wish to eat or drink; while the decorations were in keeping with all the rest. The guests were admitted into the Hall about 8 o'clock, P.M., and the feast began. But here our description must end. Prof. J. B. Johnson, of St Louis, presided, aided by the committee of arrangements. The seats at the tables were all filled, and the company seemed to enjoy, with the keenest relish, the rich repast before them.

The various *alcholic drinks* and segars, were provided in great abundance; and they were soon used freely throughout the Hall. A list of appropriate regular toasts had been provided for the occasion, and at the usual time they were read and responded to, by several of the most eminent members of the Profession. It is to be presumed that all these speeches were eloquent, truthful, and appropriate. We say *presumed*, because, though occupying a favorable position in the Hall, we really did not hear any of them with sufficient distinctness to know their merits. The President hammered the table, called to order, and entreated for silence, until he gave it up in despair.

Whoever spoke, was heard only by the few immediately around him. The confusion and noise were not owing to positive *drunkenness* and disorderly conduct; but of the 500 brains in the hall,

22

so many of them were just enough under the peculiar exhileration of wine and tobacco, to make them *too* social, hilarious, and jovial to be listening to speeches, or to be very mindful of the strict rules of order and etiquette. It was what the *world* would call *a most glorious time* ; proving most conclusively that in generous hospitality and luxurious banqueting St. Louis can rival any of the cities of Christendom. To me, however, the occasion was neither joyful nor even pleasant. Seated where I could quietly overlook the whole assembly, and having no desire to disturb the functions of the stomach by taking a variety of food at that time in the evening, and still less desire to poison my blood and irritate my brain with alcoholic beverages, or stupify it with tobacco, I had little else to do but to philosophize on the scene before me. My reflections were much influenced by the question of a learned and abstemious Judge who sat by my side as an invited guest. Soon after we were seated, and while glancing over the splendid tables before us, he modestly inquired "how much better it would be if the money invested in that banquet had been invested as a permanent fund for the aid of widows and orphans of such medical men as are cut down while devotedly striving to stay the ravages of death in the midst of pestilence.." Yes, thought I, and how much more in accordance with the beautiful motto on the wall, "the union of the Profession for the Good of Humanity." As the banqueting went on, the smoke began to curl upward from the segars, the corks popped rapidly from the wine bottles, the President called in vain for "order," ideas of bacchanalian revelry accompanied by poor-houses filled with paupers, jails filled with criminals, orphan children asking for bread, and lone sad mothers waiting at midnight for return of inebriate husbands, all came crowding on my mind, and the question whether the company before me whose professed mission was to apply all the revelations of science and the accumulated wisdom of ages to the alleviation of human suffering, was not really setting an example calculated most powerfully to encourage and sustain one of the most direful evils that ever scourged the human race, cast a shade of sadness over the mind. But aside from all reflections and reveries, is such a banquet becoming, either the dignity, the simplicity, or the scientific character of the profession? We think not; and we are sure the great mass of the profession agree with us in regard to the matter. In what we have said we cast not a word of censure on the profession of St. Louis. They only followed the example of older men in older and larger cities. But the time has come to stop. The unanimous adoption of Prof. Gross' resolution in reference to the subject, together with the well known ability and liberality of the profession in Philadelphia leaves them entirely free to set a better example at the next meeting.

Perhaps the most pleasant (in some respects) of all the items of entertainment given at St. Louis, was that furnished by the Pacific Railroad Company. The Company had extended an invitation to the Association, to make a trip over their road from St. Louis to its present western terminus, a distance of about 40 miles. This invitation was accepted and the time fixed for Friday morning, after the business of the Association had been completed At the appointed time a considerable number of Delegates, accompanied by a very agreeable company of ladies, chiefly the wives and daughters of members, were conveyed to the depot and seated in a very neat and beautiful train of cars. The route passed through a very variable section of country, passing from fields under high cultivation to scenery the most wild and romantic. The day was pleasant, the company good, the ladies especially more agreeable than ever, and the attentions of the Railroadmen all that could be desired. A very nice collation was given us at the end of the route by the Railroad Company. Here a few excellent speeches were made, and all were returned to the city safely. The Pacific Railroad Company will certainly be remembered with pleasure by all who joined in the Excursion. D.

